# Management of Facial Synkinesis with a Combination of BTX-A and Biofeedback: A Randomized Trial

**Published:** 2015-11

**Authors:** Abbas Ali Pourmomeny, Sahar Asadi, Ahmad Cheatsaz

**Affiliations:** 1 *Department of Physical Therapy, School of Rehabilitation Sciences, Isfahan University of Medical Sciences, Isfahan, Iran.*; 2 *Department of Neurology, School of Medicine, Isfahan University of Medical Sciences, Isfahan, Iran.*

**Keywords:** Bell's palsy, Biofeedback, Botulinum toxin, Synkinesis

## Abstract

**Introduction:**

Synkinesis and facial asymmetry due to facial nerve palsy are distressing conditions that affect quality of life. Unfortunately, these sequelae of facial nerve palsy are unresolved. The aim of this study was to investigate the efficacy of a combination of biofeedback therapy and botulinum toxin A (BTX-A) injection for the management of synkinesis and asymmetry of facial muscles.

**Materials and Methods:**

Among referrals from three university hospitals, 34 patients with facial synkinesis were divided randomly into two groups. All participants were evaluated using Photoshop software, videotape, and facial grading system (FGS). The first group received a single dose of BTX-A at the start of treatment, while the second group received normal saline as a control. Both groups received electromyography (EMG) biofeedback three times a week for 4 months.

**Results:**

The mean FGS values for the BTX group before and after treatment were 55.17 and 74.17, respectively, and those for the biofeedback group were 66.31 and 81.37, respectively. Moreover, it was shown that in both groups oral-ocular and oculo-oral synkinesis decreased significantly after treatment compared with before treatment (P<0.01).When these measurements were performed using Photoshop and videotape, these differences were even greater. Despite the decrease in synkinesis in both groups after treatment, there were no significant differences between the two treatment groups (P>0.05).

**Conclusion:**

Biofeedback therapy is as effective as the combination of biofeedback and BTX in reducing synkinesis and recovery of facial symmetry in Bell's palsy.

## Introduction

The incidence of peripheral facial palsy is 23–35 in 100,000 cases (1). Half of all cases of peripheral facial palsy cases are idiopathic (Bell´s palsy) and the remainder are due to tumor, trauma, injury during surgery, otitis and Ramsay Hunt syndrome (2-4). Patients exhibiting incomplete recovery of facial palsy suffer from facial muscle weakness, contracture, hyper-kinesis, hyperlacrimation, atrophy and synkinesis (1,5-7). The most debilitating sequelae of peripheral facial palsy and incomplete recovery are synkinesis and asymmetry of the face.

Synkinesis is an abnormal involuntary facial movement that occurs with a voluntary movement of a different facial muscle group (4,7-11). Synkinesis begins 3 to 4 months after regeneration of facial nerve palsy and continues for up to 2 years (6-8). The incidence of synkinesis depends on the location of facial nerve injury and is higher in intratemporal injuries than extratemporal injuries (10). Synkinesis is reported in 9–55% of patients with incomplete recovery of facial nerve palsy (7). Common types of facial synkinesis are oral-ocular synkinesis (involuntary eye closure during voluntary mouth movement) and oculo-oral synkinesis (involuntary mouth movement during voluntary eye closure) (12). The etiology of synkinesis is not fully understood. However, aberrant regeneration of facial nerves has been the most reported cause (12). Synkinesis loss of facial symmetry and unsightly appearance, drastically reduces the quality of life (13). Common interventions include surgery (neurolysis and myoctomy) and rehabilitation (8), including physical therapy modalities. Biofeedback (mirror and electromyography [EMG]) for neuromus- cular re-education has been shown to be the most effective intervention (8,9,14,15). However, biofeedback requires a long period of time and patients must make extreme efforts to achieve moderate improvement (8). 

The use of botulinum toxin type A (BTX-A ) for management of synkinesis has been studied for two decades (16). Significant relief of synkinesis may be achieved by injecting BTX-A to block the presynaptic release of acetylcholine, causing partial functional paralysis in the motor end plate (17). However, the effect of BTX-A is temporary, necessitating repeated injection and is associated with side effects, including ptosis, eye dryness, and chewing problems. Repeated BTX-A injection without biofeedback and low or high BTX-A dose with or without biofeedback have previously been recommended (18,19). Nonetheless, the most effective intervention for recovery of facial symmetry and control of synkinesis is not completely clear. Therefore, we hypothesized that a combination treatment involving BTX-A and biofeedback may be more effective than biofeedback alone in increasing facial symmetry and reducing synkinesis. This study was designed to investigate the efficacy of combining BTX-A injection and biofeedback compared with biofeedback alone in increasing facial symmetry and reducing synkinesis in patients with oral-ocular and oculo- oral synkinesis.

## Materials and Methods

A randomized clinical trial was performed in 34 patients presenting with Bell's palsy for at least 6 months together with a form of synkinesis (ocular or oral). Patients were referred from three university hospitals. Exclusion criteria included upper motor neuron facial nerve disease, keratitis, or other ocular diseases, pregnancy and neuromuscular junction disease.

The study was approved by the Committee for Medical Ethics at the Isfahan University of Medical Sciences**, **Iranian Registry of Clinical Trials (ID: IRCT201309256083N3), and informed consent was obtained from all patients before treatment. Facial features of all participants were evaluated using Adobe Photoshop software (Adobe System, Inc.) (20), videotape and facial grading system (FGS) (21). Photoshop assessment was based on a previous study. Briefly, all patients’ faces were marked and digitally photographed in four different standard expressions. Pictures were transferred to a computer and vertical axes on resting photos were determined using Adobe Photoshop. Vertical and horizontal lines were drawn in the digital picture. Then, the shortest distance from the lines to each marker was measured in the normal and impaired sides in resting and other facial expressions. Next, alteration of the marker positions was measured in millimeters using Photoshop software. 

The FGS is an observational scale, divided into three subscales that measure symmetry at rest, five standard expressions and degree of synkinesis. In the first step, the observer records the symmetry of the eye, cheek and mouth at rest; choices are provided, and given a value of 0 to 2; and the sum is multiplied by 5. The second step requires the observer to rate facial movement during five standard facial expressions on a scale of 1 to 5. Again, the values are totaled but multiplied by 4. In the third step, the level of synkinesis is evaluated on a four-point scale by the same five facial expressions as in the second step. From these three values, an overall composite score is obtained by subtraction of the synkinesis and resting score from the voluntary movement score. Overall, the assessment score is between 0 (complete paralysis) and 100 (normal).

In the videotape method, all patients were evaluated by videotaping (Canon- Digital 1xus 960 1s 12 Mega pixel) in five facial expression and rest position.

Subjects were randomly divided into two groups (BTX or biofeedback) by flipping a coin. The BTX group received BTX-A (Dysport) in combination with biofeed- back. One vial of BTX-A including 500 units was diluted with normal saline (2.5 ml) and injected into the synkinetic muscles (3–7 points) using a tuberculin syringe with a 30- gauge needle. Injection sites were orbicularis oculi, orbicularis oris, zygomatic major, levator labii superioris and depressor labii inferioris (22). As a control, normal saline solution was administered by injection in the biofeedback group at several points on the affected side. 

Two weeks after the administration of BTX-A or saline, both groups initiated rehabilitation that included stretching the muscles of the affected side and EMG biofeedback using an EB Neuro-MYTO II instrument which has two channels (one for voluntary movement and the other for involuntary movement). During the treatment period, patients received three sessions per week of approximately 30-minute duration and performed mirror biofeedback as home training. After 4 months of treatment, patients were re-evaluated using Photoshop, videotape and FGS; as before treatment. Videotapes were evaluated by three professional therapists who were blinded to the information regarding patient group or treatment status. Synkinesis was rated as absent, mild, moderate, or severe. Photoshop assessment and FGS were evaluated by one researcher who was blinded to treatment groups. 

The results of the three evaluation methods were analyzed by t-test, paired t-test, Mann-Whitney and Wilcoxon tests (α=0.05).

## Results

Twenty-eight female and four male patients with Bell's palsy were recruited into the study. Demographic data of the patients are given in (Table.1). No participants reported complications after injection. No significant differences between the three evaluation methods were found when the entire study sample was analyzed.

**Table 1:** Patient characteristics according to Bell's palsy

**Demographic group**	**BTX-A + biofeedback**	**Biofeedback**
Mean age (yrs.)	36.11	39.11
Sex:		
Female	12	16
Male	5	1
Side:		
Right	9	6
Left	8	11
Duration; (means ±SD years)	5±6.93	3±40
Ocular & oral synkinesis	17	17

***FGS***

Statistical tests showed that the mean change in FGS was 19% in the BTX group and 15% in the biofeedback group. Differences between values before and after treatment were significant for both groups (P<0.05) (Table. 2). Mean change in FGS pre- and post-treatment were 15.6 in the BTX group and 11.9 in the biofeedback group. The differences between post-treatment values of the two groups were not significant (P>0.05) (Table. 3).

**Table 2:** Facial grading system and Photoshop results (pre- and post-treatment) in ocular and oral synkinesis

		**FGS**		**Photo Shop**				
				**Ocular synkinesis**		**Oral synkinesis**		
Group	N	Before treatment (mean ± SD)	After treatment (mean ± SD)	Before treatment (mean ± SD)	After treatment (mean ± SD)	Before treatment (mean ± SD)	After treatment (mean ± SD)	p
Botox	17	55.17±19	74.17±13	17.5±8	6.9±4	40.7±14	27.4±16	<0.05
Biofeedback	17	66.31±17	81.37±11	22.7±5	10.8±3	39.2±8	26.3±7	<0.05
p-value		>0.05	>0.05	>0.05	>0.05	>0.05	>0.05	

FGS: Facial grading system, SD: standard deviation

**Table 3:** Mean differences pre- and post-treatment according to facial grading system and Photoshop

**Group** **FGS**		**Botox**	**Biofeedback**	**P**
	Change: pre & post-treatment (mean ± SD)	15.6±13	11.9±11	>0.05
Photoshop scale	Change: pre & post-treatment (mean ± SD) (Ocular synkinesis)	10.5±4	11.8±5	>0.05
	Change: pre & post-treatment (mean ± SD) (Oral synkinesis)	13.2±10	13.3±4	>0.05

FGS: Facial grading system, SD: standard deviation

***Photoshop***

The mean Photoshop values in ocular synkinesis for the BTX group before and after treatment were 17.5 and 6.9, respectively, and those for the biofeedback group were 22.7 and 10.8, respectively. Also, mean Photoshop values in oral synkinesis for the BTX group before and after treatment were 40.7 and 27.4, respectively, and those for the biofeedback group before and after treatment were 39.2 and 26.3, respectively (Table.2). These differences were statistically significant (P<0.05). However, mean differences in ocular and oral synkinesis, determined using Photoshop were not significant between groups after treatment (P>0.05) (Table.3).

***Video tape***

Oral and ocular synkinesis was determined by videotape. Oral synkinesis decreased in 17 patients and increased in four patients; no changes were observed in 13 patients. Ocular synkinesis decreased in 14 patients and increased in one patient; no changes were observed in 19 patients. Score changes of both groups are given in (Table.4). Both oral and ocular synkinesis showed significant changes after treatment (P<0.05). In contrast, no significant differences in synkinesis type between the two groups after treatment was found (P>0.05).

**Table 4:** Change in oral and ocular synkinesis by evaluation videotape

**Group**	**Oral synkinesis**			**Ocular synkinesis**		
	Decrease	Increase	Without change	Decrease	Increase	Without change
Biofeedback	8	2	7	6	0	11
BTX	9	2	6	8	1	8
Total	17	4	13	14	1	19
P-value	>0.05			>0.05		


## Discussion

In this study, asymmetry and synkinesis evaluation using Photoshop, FGS and videotape were performed before and after regular neuromuscular re-education with biofeedback in two groups. The methods were independent of each other and were blinded. Both groups exhibited improvement in the symmetry of facial movements, voluntary facial movements and control of synkinesis. In all these three methods, the results were the same and confirm the efficacy of biofeedback therapy.

There are a limited number of clinical trials and research focused towards the efficacy of biofeedback therapy and BTX to treat sequelae facial nerve palsy (8,18,19). A comparison between the use of Kabat exercise or proprioceptive neuromuscular facilitation (PNF) alone and the combination of BTX and PNF with low sample size was reported in 2011 by Monini et al.(23). Patients graded House-Brackmann II and III were enrolled in this study and were treated by PNF and evaluated by partial FGS. However, asymmetry of the face was not reported as an outcome in this study. In contrast to our study, Monini et al. showed that BTX as a complementary therapy could help reduce synkinesis. In our study, in addition to reducing synkinesis, the impact on facial symmetry and muscle tension were also considered.

 Unlike skeletal muscles, facial muscles are small and delicate with minimal contraction, and at least one connection (insertion) ends at the fascia or skin(24-26). Therefore, facial muscles are predisposed to contracture and deformity. For these reasons, facial nerve palsy sequelae are not limited to synkinesis. In addition to muscle weakness, tension and contracture, they impact coordination between the muscles of each side and result in asymmetry between the two sides of the face. 

Therefore, neuromuscular facial retraining using EMG biofeedback is a preferred modality and is very effective in increasing functional facial movements and inhibiting synkinesis. It is a particularly important part of the program because it provides immediate feedback to the patient, as well as providing an objective means of measuring movements and outcomes. It might be possible that synkinesis is relieved temporarily with a few BTX injections. This means that all motor units in the site of injection are suppressed temporarily or permanently, with some motor units destroyed and part of the muscle paralyzed and atrophied. However, the subsequent biophysical processes, possibly involving a sprouting mechanism or mass movement, remain unclear. It is known, however, that BTX injection may leave patients without correction of asymmetry, as well as with mass movement asymmetry and increasing synkinesis (27). Therefore, movement sequelae in facial nerve palsy first require a decrease in muscle tension and contracture (26,27). Then each facial expressions should be analyzed, weakened muscles identified and compared with the unaffected side, and finally a tailored method selected for patient management. Neuromuscular re-education based on brain neuroplasticity could be an appropriate method (6). However, this method should be proportional according to the limitations of proprioception in the facial muscles (25).Therefore EMG biofeedback, requiring considerable skill compared with exercise therapy, could be a suitable method and an effective instrument in controlling synkinesis and creating coordination between the two sides of the face (14,28,29). In future studies, we would recommend comparing the direct influence of BTX and biofeedback therapy on the sequelae of facial nerve palsy, as well as evaluating the effect when BTX is injected directly into the muscle. Because of time constraints in this study, we were not able to follow the patients for more than 4 months.

## Conclusion

This study showed that neuromuscular re-education accomplished using EMG biofeedback with or without BTX-A injection could control synkinesis and improve coordination of movements on both sides of the face.
